# A Combination of Extended Fuzzy AHP and Fuzzy GRA for Government E-Tendering in Hybrid Fuzzy Environment

**DOI:** 10.1155/2014/123675

**Published:** 2014-06-26

**Authors:** Yan Wang, Chengyu Xi, Shuai Zhang, Dejian Yu, Wenyu Zhang, Yong Li

**Affiliations:** School of Information, Zhejiang University of Finance and Economics, Hangzhou 310000, China

## Abstract

The recent government tendering process being conducted in an electronic way is becoming an inevitable affair for numerous governmental agencies to further exploit the superiorities of conventional tendering. Thus, developing an effective web-based bid evaluation methodology so as to realize an efficient and effective government E-tendering (GeT) system is imperative. This paper firstly investigates the potentiality of employing fuzzy analytic hierarchy process (AHP) along with fuzzy gray relational analysis (GRA) for optimal selection of candidate tenderers in GeT process with consideration of a hybrid fuzzy environment with incomplete weight information. We proposed a novel hybrid fuzzy AHP-GRA (HFAHP-GRA) method that combines an extended fuzzy AHP with a modified fuzzy GRA. The extended fuzzy AHP which combines typical AHP with interval AHP is proposed to obtain the exact weight information, and the modified fuzzy GRA is applied to aggregate different types of evaluation information so as to identify the optimal candidate tenderers. Finally, a prototype system is built and validated with an illustrative example for GeT to confirm the feasibility of our approach.

## 1. Introduction

The basic principles of the tendering process have been applied to many business areas, such as purchasing goods, seeking service providers, business consulting, or the selection of main contractors for construction work [1]. Meanwhile, tendering has also been widely applied to government affairs for the obvious fairness generated by tendering. However, along with the fairness, traditional tendering process may bring some practical issues such as low efficiency, high cost and insufficient transparency, and accountability. Additionally, traditional tendering process is paper-based and involves much manual work, which can also cause many problems [2].

Therefore, in order to solve the above problems, researchers have introduced the E-tendering system [2–6]. As a combination of conventional tendering and Internet technologies, computer technologies, certification technologies, and so forth, E-tendering is much more efficient, transparent, and accountable than conventional tendering process for its eminent nature. Furthermore, government E-tendering (GeT) can (1) reduce the costs of both tenderees and tenderers and improve the efficiency of tendering procedures greatly, (2) eliminate paper work and invest less labor and resources into tender document preparation, and (3) standardize market order, suppress bid collusion, establish a fairer and more transparent tendering environment, and so forth.

Developing and promoting an efficient and effective government E-tendering system so as to further optimize the conventional government tendering process is a complicated project that contains numerous subsystems. Web-based bid evaluation system is a crucial one of those subsystems, which aims at identifying the optimal tenderer with the given information of tenderers using efficient and effective methodologies or methods. In this paper, we will firstly investigate the potentiality of a combined methodology, which is a combination of extended fuzzy analytic hierarchy process (AHP) and modified fuzzy gray relational analysis (GRA), to meet the demands of government E-tendering. The novel hybrid fuzzy AHP-GRA (HFAHP-GRA) methodology is proposed in a hybrid fuzzy environment, where the information of tenderers is expressed as four different types of numbers (real number, interval number, triangular number, and intuitionistic fuzzy number) with consideration of a reality that experts are most likely to express their evaluations on tenderers as different types of numbers. Compared with typical AHP and interval AHP, the extended fuzzy AHP can deal with interval preference matrices while typical AHP cannot. The extended fuzzy AHP can also obtain the exact weight information of alternatives while interval AHP cannot. The modified fuzzy GRA, rather than typical fuzzy GRA, can further aggregate four different types of evaluation information in one evaluation matrix.

The remainder of this paper is arranged as follows. Some related works are discussed in Section 2. The HFAHP-GRA methodology will be elaborated in Section 3, including an extended fuzzy AHP, the fuzzy GRA algorithm, and some related formulations. A prototype system for government E-tendering based on our proposed methodology will be illustrated in Section 4. In Section 5, conclusions will be discussed.

## 2. Related Works

### 2.1. Government E-Tendering

The past decades have seen the rapid development of Internet technologies, communication technologies, computer technologies, certification technologies, and so forth. These technologies make it possible to realize the electronization and informatization of conventional tendering process. Both public and private sectors in various business categories agree that efficiencies can be made through the use of E-procurement whilst maintaining quality and producing greater value-for-money [7]. As the fairest E-procurement method, E-tendering's high efficiency and obvious cost saving have successfully made an increasing number of governmental agencies aware of the importance of it.

However, the uptake of E-tendering in numerous governmental agencies has been slower than expected despite the fact that E-procurement systems have already been widely applied in many countries. The situation is that most systems are only used for providing procurement information, receiving bidding information and venders' catalogs, and using purchase cards on procurement of small items [8]. In particular, existing government E-tendering systems cannot be able to deal with vague, uncertain, and incomplete tendering information. Thus, the superiorities of government E-tendering cannot be exploited to the full extent. Therefore, developing an efficient and effective government E-tendering system that is able to deal with fuzzy tendering information is necessary and imperative.

Web-based bid evaluation system is a crucial part of the whole government E-tendering system, which has been applied to identify the optimal tenderer given the information of different tenderers by means of Internet or artificial intelligence technologies. It aims at replacing traditional manual bid evaluation process so as to suppress bid collusion, develop efficiency, and save costs. Since traditional bid evaluation system can hardly satisfy modern bid evaluation because of the explosion of information and the uncertainty, vagueness, dynamicity, and complication of current bid evaluation environment, it is reasonable to develop an efficient and effective web-based bid evaluation system. Singh and Benyoucef [9] have presented a TOPSIS-based bid methodology for e-sourcing to solve the sealed bid, multiattribute reverse auction problem. Yan et al. [10] have developed a web-based decision support system that synthetically applies four different evaluation methods to comprehensive bid evaluation of agricultural machinery. Bar et al. [11] have proposed an experience-based evaluation process for Enterprise Resource Planning (ERP) bids. Wang et al. [12] have proposed a modified bid evaluation mechanism to diminish the potential antagonism between technical and business experts in online procurement auctions.

However, restricted by their theoretical assumptions and mechanisms, the above systems are unable to adapt to a vague, uncertain, complicated, and dynamic tendering circumstance; that is, these systems are not suitable for government E-tendering. In this paper, we will firstly apply the combination of extended fuzzy AHP and modified fuzzy GRA, namely, HFAHP-GRA methodology, for government E-tendering. The whole HFAHP-GRA methodology is proposed in a hybrid fuzzy circumstance, where the evaluations of experts on tenderers' attributes are expressed as different kinds of numbers, such as real number, triangular number, intuitionistic fuzzy number, and interval number. Additionally, we assume that both weight information of experts and attributes of tenderers are incompletely known. Thus, the evaluation environment can be described as accurately and objectively as possible during evaluation process.

### 2.2. Hybrid Fuzzy Information

There exist two kinds of concepts: clear concept and fuzzy concept. Clear concept refers to concepts that are certain, definite, and specific, such as “tree” and “flower.” On the contrary, fuzzy concept refers to those concepts that are uncertain, indefinite, and abstract, such as “good” and “beautiful.” In fact, fuzzy concepts are much more common. The common mathematical models are not able to deal with those fuzzy concepts because of their natures from birth.

Thus, exploring new mathematical theories to bridge the gap between mathematics and fuzzy concepts is imperative. Zadeh [13] firstly proposed the fuzzy set theory in 1965. With the fuzzy set theory as the theory base, the theoretical foundation of fuzzy mathematics has been set up gradually so that decision makers can apply quantity relations to describe fuzzy concepts and make fuzzy operations. In 1975, Zadeh [14] further proposed and explored the linguistic variable whose values are words or sentences in a natural or artificial language. This theory has made great contribution to numerous areas, such as artificial intelligence and automatic control. In 1986, Atanassov [15] extended the fuzzy set theory and introduced the concept of intuitionistic fuzzy set (IFS). Thereafter, many efforts [16–20] have been taken to further improve and extend the IFS theory. Interval number, as a special form of fuzzy number, has already been applied to fuzzy decision making decades ago. Moore et al. [21] introduced the basic terms and concepts of the interval number and explored the operations of interval arithmetic and further extended the interval theory.

However, only using one of those different kinds of fuzzy numbers to describe evaluation information or attribute information is insufficient for government E-tendering, because the web-based bid evaluation process involved is complicated and comprehensive. What is worse, the evaluation environment is uncertain, vague, and dynamic. Therefore, Xu [22] proposed a dynamic geometric aggregation operator, which applies three representation formats, say, real number, interval number, and triangular number (triangular numbers are used to transform linguistic labels), for dynamic hybrid multiattribute group decision making (DHMADM). Then, Wei [23] proposed a GRA based dynamic geometric aggregation operator applying the same three representation formats for DHMADM. However, these two operators have some difficulties in dealing with DHMADM during which the weight information of experts or tenderer's attributes is incompletely known.

Therefore, in this paper, we propose the extended fuzzy AHP to deal with the above problem. Additionally, we use four representation formats, including real number, interval number, triangular number (that are used to transform linguistic labels), and intuitionistic fuzzy number, to describe evaluation information and attribute information. This aims at expressing related bid evaluation information more objectively, authentically, and comprehensively.

### 2.3. Analytic Hierarchy Process

**Figure 1:**
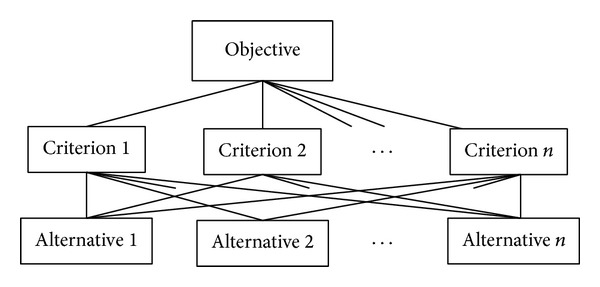
The structure of typical AHP.

Saaty [24] is believed to be the first researcher who proposed AHP, which has been widely applied to numerous industries [25–30].  Figure 1 displays the structure of a typical AHP. Generally, it has three levels: objective level, criteria level, and alternative level.

The basic idea of typical AHP is based on the pairwise comparison matrices. Each element of a matrix stands for the personal preference of decision maker on one alternative versus another one, which is usually expressed as linguistic terms. These linguistic terms can then be transformed into Likert numbers from one to nine or decimal numbers between 0 and 1. Consistence check of comparison matrix is realized by a consistency ratio *CI*.

Though typical AHP is a convenient, flexible, and effective multicriteria decision making approach that combines qualitative analysis with quantitative analysis, it still has shortages in dealing with the transformation of qualitative information into quantitative information. Likert numbers are discrete and dispersive, while the preferences of decision maker are consecutive. Therefore, the theoretical assumption of the transformation of decision maker's preferences into Likert numbers is defective. In order to alleviate such deficiency, we choose to translate preferences into interval Likert numbers so as to make the translation as reasonable as possible. After preferences are transformed into interval Likert number, singly typical AHP will be no longer available. Thus, we proposed an extended fuzzy AHP which combines typical AHP theory with interval AHP theory in this paper.

### 2.4. Gray Relational Analysis

**Figure 2:**
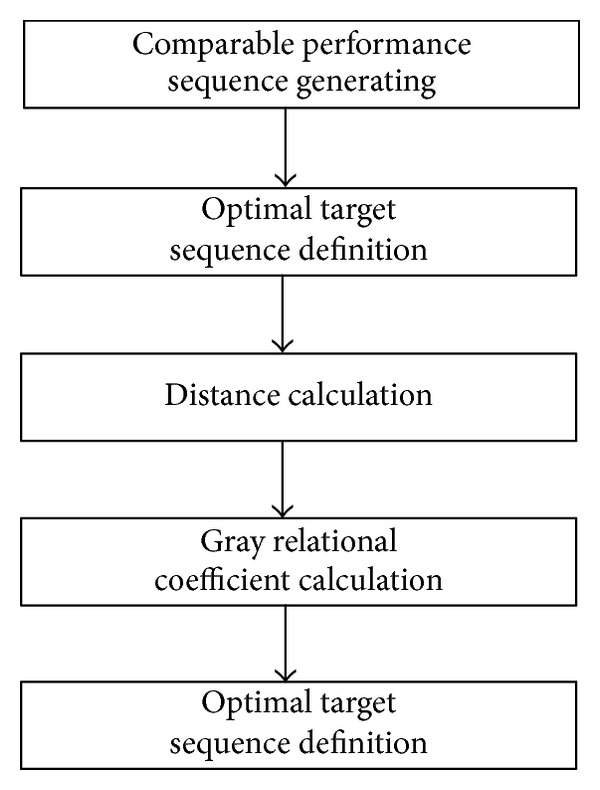
The basic process of GRA.

GRA method was originally proposed by Deng [31] and has been successfully applied to many fields [32–37]. The first step of the main process of GRA is normalizing the performance of alternatives so as to generate comparable performance sequences of alternatives. Then, according to the performance sequences, the optimal target sequence can be defined. Each sequence including performance sequence and target sequence consists of *n* values if there exist *n* evaluation criteria. Thus, the distance between each performance value and the optimal target value can be calculated. Thereafter, the gray relational coefficient between each performance sequence and the optimal target sequence is obtained. Finally, the gray relational grade between each performance sequence and the optimal target sequence can be calculated according to those gray relational coefficients. Through ranking the alternatives based on the value of gray relational grade, one can obtain the optimal alternative. The basic process of GRA is shown in Figure 2.

Generally, the elements of evaluation matrix of one GRA process are always expressed as values sharing the same date type. However, in this paper, the evaluation matrices consist of four different data types: real number, interval number, triangular number, and intuitionistic fuzzy number. Therefore, according to the different data types, we need to apply correspondingly different methods to realize the normalization and distance calculation during GRA process.

## 3. A Combined Methodology for Government E-Tendering

The integrated AHP-GRA method has already been widely researched and applied to many areas [38–44], such as portfolio investment in stock market [38], supplier selection [39, 40], and tannery effluent treatment [41]. Some researchers further explored the application of fuzzy AHP-GRA method [45–47]. This method solves the problem when the evaluation process is too subjective and effectively compensates for the lack of establishing the weight [45]. It is found that the application of fuzzy AHP-GRA method can increase the reliability and accuracy of the evaluation results. However, those applications of fuzzy AHP-GRA still have a common drawback; that is, the way of describing attribute information is too simplex to quantify attribute information as objective, authentic, and comprehensive as possible.

This paper will firstly investigate the potentiality of a novel HFAHP-GRA methodology. Four different types of fuzzy numbers (real number, triangular number, intuitionistic fuzzy number, and interval number) will be used to describe bid evaluation information so as to ensure the objectivity, authenticity, and comprehensiveness of the quantification process of bid evaluation information. The proposed novel HFAHP-GRA methodology consists of two main stages: weight information obtaining and optimal tenderer identification.

### 3.1. Using Extended Fuzzy AHP to Obtain the Weight Information of Experts and Evaluation Criteria

**Figure 3:**
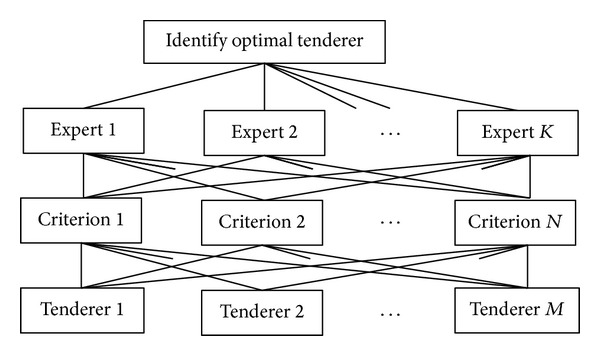
The structure of extended fuzzy AHP.

Mostly, a decision maker cannot exactly express his/her personal preference on one alternative versus another one. In this paper, we assume a decision maker expresses his/her opinions by means of an interval multiplicative preference comparison matrix (IMPCM). Besides, different from the typical AHP, the extended fuzzy AHP in this paper has four levels including objective level, expert level, criteria level, and alternative level, shown in Figure 3.

Let *u*_*ij*_ = [*u*_*ij*_^−^, *u*_*ij*_^+^] (*i* = 1,2,…, *K*, *j* = 1,2,…, *K*) be the interval preference of government tendering sector on expert *i* versus expert *j*, and let *v*_*ijk*_ = [*v*_*ijk*_^−^, *v*_*ijk*_^+^] (*i* = 1,2,…, *N*, *j* = 1,2,…, *N*, *k* = 1,2,…, *K*) be the interval preference of expert *k* on evaluation criterion *i* versus evaluation criterion *j*. Then, we can obtain the IMPCM *U* of government tendering sector on experts and the IMPCM *V*_*k*_ of expert *k* on evaluation criteria, shown as follows:

(1) $$\begin{array}{c} U=\left[\begin{array}{cccc} \left[1,1\right] & \left[u_{12}^{-},u_{12}^{+}\right] & \cdots & \left[u_{1K}^{-},u_{1K}^{+}\right]\\ \left[u_{21}^{-},u_{21}^{+}\right] & \left[1,1\right] & \cdots & \left[u_{2K}^{-},u_{2K}^{+}\right]\\ \cdots & \cdots & \cdots & \cdots \\ \left[u_{K1}^{-},u_{K1}^{+}\right] & \left[u_{K2}^{-},u_{K2}^{+}\right] & \cdots & \left[1,1\right] \end{array}\right],\\ V_{k}=\left[\begin{array}{cccc} \left[1,1\right] & \left[v_{12k}^{-},v_{12k}^{+}\right] & \cdots & \left[v_{1Nk}^{-},v_{1Nk}^{+}\right]\\ \left[v_{21k}^{-},v_{21k}^{+}\right] & \left[1,1\right] & \cdots & \left[v_{2Nk}^{-},v_{2Nk}^{+}\right]\\ \cdots & \cdots & \cdots & \cdots \\ \left[v_{N1k}^{-},v_{N1k}^{+}\right] & \left[v_{N2k}^{-},v_{N2k}^{+}\right] & \cdots & \left[1,1\right] \end{array}\right], \end{array}$$

where *u*_*ij*_^−^, *u*_*ij*_^+^, *v*_*ijk*_^−^, and *v*_*ijk*_^+^ are Likert numbers, 1/9 ≤ *u*_*ij*_^−^ ≤ *u*_*ij*_^+^ ≤ 9, 1/9 ≤ *v*_*ijk*_^−^ ≤ *v*_*ijk*_^+^ ≤ 9, *u*_*ij*_^−^ · *u*_*ij*_^+^ = 1, and *v*_*ijk*_^−^∗*v*_*ijk*_^+^ = 1.

The consistency and acceptable consistency of a real-numbered multiplicative preference comparison matrix (RMPCM) have been defined by Saaty [24]. For a RMPCM, if its consistency ratio *C*.*R*. is 0.1 or less, we can consider that this RMPCM is the acceptable consistency. The expression of *C*.*R*. is presented below:

(2) $$\begin{array}{c} C.R.=\frac{C.I.}{R.I.},\\ C.I.=\frac{\lambda_{\max}-n}{n-1}, \end{array}$$

where *n* and *λ*_max⁡_ are the number of dimensionality and the largest eigenvalue of RMPCM, respectively, and *R*.*I*. is the average of randomly generated *C*.*I*., which depends on *n*.  Table 1 [24] shows the standard values of *R*.*I*.

However, the above definition is not applicable for IMPCM. Liu [48] addressed the consistency and acceptable consistency of IMPCM. Hereafter, we take *U* as an example to show the related definitions and expressions. Let *B* = (*b*_*ij*_)_*n*×*n*_ and *C* = (*c*_*ij*_)_*n*×*n*_, where

(3) $$\begin{array}{c} b_{ij}=\{\begin{array}{cc} u_{ij}^{+}, & i<j\\ 1, & i=j,\\ u_{ij}^{-}, & i>j, \end{array}\\ c_{ij}=\{\begin{array}{cc} u_{ij}^{-}, & i<j\\ 1, & i=j,\\ u_{ij}^{+}, & i>j. \end{array} \end{array}$$

Then, *B* and *C* are multiplicative preference comparison matrices. According to Liu [48], only if both *B* and *C* are consistent or acceptably consistent, *U* is said to be consistent or acceptably consistent.

Therefore, by using expressions (1)–(3), the acceptable consistency of *U* can be checked. Similarly, the acceptable consistency of *V*_*k*_ can also be checked. If *U* and *V*_*k*_ are unacceptably consistent, the consistency improving method proposed by Xu and Wei [49] can be used to further improve the consistency of *U* and *V*_*k*_.

After checking the acceptable consistency of *U* and *V*_*k*_, the following expression is further applied to obtain the interval weight vector of *U* [48]:

(4) ωi=[min⁡{(∏j=1Kbij)1/K,(∏j=1Kcij)1/K},max⁡{(∏j=1Kbij)1/K,(∏j=1Kcij)1/K}],

where *ω*_*i*_ is the interval weight of expert *i*. Similarly, the interval weight vector of *V*_*k*_ can be yielded. Thus, the interval weight vector *ω* = {[*ω*_1_^*L*^, *ω*_1_^*H*^], [*ω*_2_^*L*^, *ω*_2_^*H*^],…, [*ω*_*K*_^*L*^, *ω*_*K*_^*H*^]} of *U* and the interval weight vector *ω*_*k*_ = {[*ω*_1*k*_^*L*^, *ω*_1*k*_^*H*^], [*ω*_2*k*_^*L*^, *ω*_2*k*_^*H*^],…, [*ω*_*NK*_^*L*^, *ω*_*NK*_^*H*^]} of *V*_*k*_ are obtained.

According to the ranking principles of two interval weights (let *ω*_*i*_ = [*ω*_*i*_^*L*^, *ω*_*i*_^*H*^] and *ω*_*j*_ = [*ω*_*j*_^*L*^, *ω*_*j*_^*H*^]) proposed by Liu [48], if *ω*_*i*_^*L*^ = *ω*_*i*_^*H*^ and *ω*_*j*_^*L*^ = *ω*_*j*_^*H*^, we have

(5) $$\begin{array}{c} p\left(\omega_{i}\geq \omega_{j}\right)=\{\begin{array}{cc} 1 & \text{if}\;\;\omega_{i}>\omega_{j},\\ \frac{1}{2} & \text{if}\;\;\omega_{i}=\omega_{j},\\ 0 & \text{if}\;\;\omega_{i}<\omega_{j}. \end{array} \end{array}$$

If *ω*_*i*_^*L*^ = *ω*_*i*_^*H*^ = *ω*_*i*_ and *ω*_*j*_^*L*^ ≠ *ω*_*j*_^*H*^, we have

(6) $$\begin{array}{c} p\left(\omega_{i}\geq \omega_{j}\right)=\{\begin{array}{cc} 1 & \text{if}\;\;\omega_{i}>\omega_{j}^{H},\\ \frac{\omega_{i}-\omega_{j}^{L}}{\omega_{j}^{H}-\omega_{j}^{L}} & \text{if}\;\;\omega_{j}^{L}\leq \omega_{i}\leq \omega_{j}^{H},\\ 0 & \text{if}\;\;\omega_{i}<\omega_{j}^{L}. \end{array} \end{array}$$

If *ω*_*i*_^*L*^ ≠ *ω*_*i*_^*H*^ and *ω*_*j*_^*L*^ = *ω*_*j*_^*H*^ = *ω*_*j*_, we have

(7) $$\begin{array}{c} p\left(\omega_{i}\geq \omega_{j}\right)=\{\begin{array}{cc} 1 & \text{if}\;\;\omega_{i}^{L}>\omega_{j}^{},\\ \frac{\omega_{i}^{H}-\omega_{j}^{}}{\omega_{i}^{H}-\omega_{i}^{L}} & \text{if}\;\;\omega_{i}^{L}\leq \omega_{j}\leq \omega_{i}^{H},\\ 0 & \text{if}\;\;\omega_{i}^{H}<\omega_{j}^{}. \end{array} \end{array}$$

**Figure 4:**
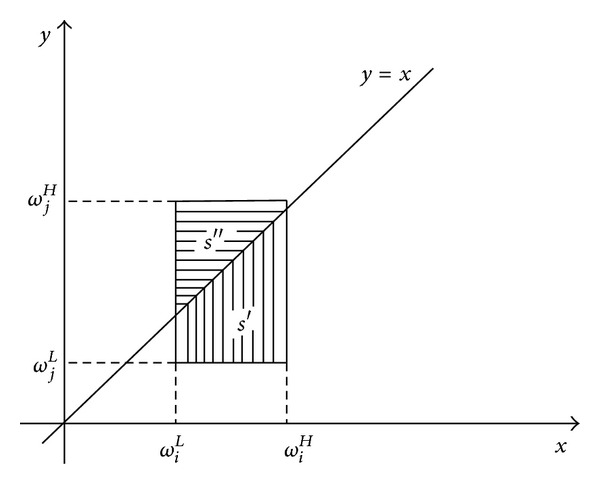
A two-dimensional style of two interval weights.

If *ω*_*i*_^*L*^ ≠ *ω*_*i*_^*H*^ and *ω*_*j*_^*L*^ ≠ *ω*_*j*_^*H*^, we have

(8) $$\begin{array}{c} p\left(\omega_{i}\geq \omega_{j}\right)=\frac{s^{\prime }}{s},\\ p\left(\omega_{j}\geq \omega_{i}\right)=\frac{s^{\prime \prime }}{s}, \end{array}$$

where *s* = (*ω*_*i*_^*H*^ − *ω*_*i*_^*L*^)(*ω*_*j*_^*H*^ − *ω*_*j*_^*L*^), *s*′ + *s*′′ = *s*, and *s*′ and *s*′′ are shown in Figure 4 [48].

Thus, the possibility degree matrices *P* = (*p*_*ij*_)_*K*×*K*_ and *P*_*k*_ = (*p*_*ijk*_)_*N*×*N*_ can be obtained, which just satisfy the definition of additive preference comparison matrix [50–52]; that is, *P* and *P*_*k*_ are additive preference comparison matrices. As the current situation, almost all the state-of-the-art literatures applied a row-column elimination method to generate a ranking vector from the possibility degree matrix. This practice is available in ranking alternatives as opposed to obtaining the exact weight information of alternatives. Thus, in order to get the exact weight information of experts and evaluation criteria, we need to utilize another crisp-valued AHP process. The first few steps are the same as the ones mentioned above, while the last three steps that have not been mentioned yet are shown as follows.

(1) Transforming additive preference comparison matrix into multiplicative preference comparison matrix. Liu et al. [50] proposed a transformation formula for additive preference comparison matrix and multiplicative preference comparison matrix, shown as follows:

(9) $$\begin{array}{c} p_{ij}^{\prime }=9^{2p_{ij}-1}, \end{array}$$

where *p*_*ij*_′ is an element of multiplicative preference comparison matrix *P*′ = (*p*_*ij*_′)_*K*×*K*_. Hence, additive preference comparison matrices *P* and *P*_*k*_ can be transformed into multiplicative preference comparison matrices *P*′ = (*p*_*ij*_′)_*K*×*K*_ and *P*_*k*_′ = (*p*_*ijk*_′)_*N*×*N*_.

(2) Since the consistency of *P*′ and *P*_*k*_′ has been checked, the eigenvalues of *P*′ and *P*_*k*_′ need to be calculated.

(3) Then, by normalizing the eigenvectors corresponding to the largest eigenvalues, the weight information of experts and evaluation criteria, which is presented as crisp values, can be obtained.

### 3.2. Using Modified Fuzzy GRA to Identify the Optimal Tenderer

The attribute information generally consists of two types: real numbers and linguistic terms. For example, price is always represented in the format like real number or interval number, while most of the other attributes, such as feasibility, artistry, and functionality,, are shown as linguistic terms like “bad,” “medium,” “good,” and so forth. Therefore, quantifying these linguistic terms reasonably and effectively is a very important job. In this paper, we apply four representation formats, say, real number, interval number, triangular number, and intuitionistic fuzzy number, to enhance the reasonability and effectiveness of quantification process. Wei [23] has displayed an effective method (shown in Table 2) for transforming linguistic terms into triangular numbers, which is adopted in this work. Accordingly, the evaluation matrix *A*_*k*_ of expert *k* with quantified attribute information is shown as follows:

(10) $$\begin{array}{c} A_{k}=\left[\begin{array}{ccccc} a_{11k} & \left[b_{12k}^{-},b_{12k}^{+}\right] & \left(c_{13k}^{L},c_{13k}^{M},c_{13k}^{H}\right) & \cdots & \left\{\left[d_{1Nk}^{M-},d_{1Nk}^{M+}\right],\left[d_{1Nk}^{N-},d_{1Nk}^{N+}\right]\right\}\\ a_{21k} & \left[b_{22k}^{-},b_{22k}^{+}\right] & \left(c_{23k}^{L},c_{23k}^{M},c_{23k}^{H}\right) & \cdots & \left\{\left[d_{2Nk}^{M-},d_{2Nk}^{M+}\right],\left[d_{2Nk}^{N-},d_{2Nk}^{N+}\right]\right\}\\ a_{31k} & \left[b_{32k}^{-},b_{32k}^{+}\right] & \left(c_{33k}^{L},c_{33k}^{M},c_{33k}^{H}\right) & \cdots & \left\{\left[d_{3Nk}^{M-},d_{3Nk}^{M+}\right],\left[d_{3Nk}^{N-},d_{3Nk}^{N+}\right]\right\}\\ \cdots & \cdots & \cdots & \cdots & \cdots \\ a_{M1k} & \left[b_{M2k}^{-},b_{M2k}^{+}\right] & \left(c_{M3k}^{L},c_{M3k}^{M},c_{M3k}^{H}\right) & \cdots & \left\{\left[d_{MNk}^{M-},d_{MNk}^{M+}\right],\left[d_{MNk}^{N-},d_{MNk}^{N+}\right]\right\} \end{array}\right], \end{array}$$

where *a*_*ijk*_, [*b*_*ijk*_^−^, *b*_*ijk*_^+^], (*c*_*ijk*_^*L*^, *c*_*ijk*_^*M*^, *c*_*ijk*_^*H*^), and {[*d*_*ijk*_^*M*−^, *d*_*ijk*_^*M*+^], [*d*_*ijk*_^*N*−^, *d*_*ijk*_^*N*+^]} (*i* = 1,2,…, *M*, *j* = 1,2,…, *N*, *k* = 1,2,…, *K*) are real number, interval number, triangular number, and interval-valued intuitionistic fuzzy number, respectively. They are different types of evaluation information of expert *k* on criterion *j* of tenderer *i*. Thus, we call *A*_*k*_ a hybrid fuzzy evaluation matrix (HFEM).

The following steps display the process of using modified fuzzy GRA to identify the optimal tenderer.

*(a) Evaluation Value Normalization*. The evaluation value of expert *k* on each evaluation criterion of each tenderer needs to be normalized; that is, the hybrid fuzzy evaluation matrix *A*_*k*_ needs to be normalized into the matrix *R*_*k*_. With consideration of the evaluation criteria consisting of benefit criteria and cost criteria, their normalization methods are a little different. Wei [23] has proposed the normalization methods for real number, interval number, and triangular number, while interval-valued intuitionistic fuzzy number has already been normalized from the very beginning. The following shows the normalization methods.

For benefit attributes, consider

(11) $$\begin{array}{c} a_{ijk}^{\prime }=\frac{a_{ijk}^{}}{\sum_{i=1}^{M}a_{ijk}^{}},\\ b_{ijk}^{\prime }=\left[\frac{b_{ijk}^{-}}{\sum_{i=1}^{M}b_{ijk}^{+}},\frac{b_{ijk}^{+}}{\sum_{i=1}^{M}b_{ijk}^{-}}\right],\\ c_{ijk}^{\prime }=\left[\frac{c_{ijk}^{L}}{\sum_{i=1}^{M}c_{ijk}^{H}},\frac{c_{ijk}^{M}}{\sum_{i=1}^{M}c_{ijk}^{M}},\frac{c_{ijk}^{H}}{\sum_{i=1}^{M}c_{ijk}^{L}}\right], \end{array}$$

where *i* = 1,2,…, *M*, *j* = 1,2,…, *N*.

For cost attributes, consider

(12) $$\begin{array}{c} a_{ijk}^{\prime }=\frac{1/a_{ijk}^{}}{\sum_{i=1}^{M}1/a_{ijk}^{}},\\ b_{ijk}^{\prime }=\left[\frac{1/b_{ijk}^{+}}{\sum_{i=1}^{M}1/b_{ijk}^{-}},\frac{1/b_{ijk}^{-}}{\sum_{i=1}^{M}1/b_{ijk}^{+}}\right],\\ c_{ijk}^{\prime }=\left[\frac{1/c_{ijk}^{H}}{\sum_{i=1}^{M}1/c_{ijk}^{L}},\frac{1/c_{ijk}^{M}}{\sum_{i=1}^{M}1/c_{ijk}^{M}},\frac{1/c_{ijk}^{L}}{\sum_{i=1}^{M}1/c_{ijk}^{H}}\right], \end{array}$$

where *i* = 1,2,…, *M*, *j* = 1,2,…, *N*.

*(b) Ideal Tenderer Definition*. Defining the ideal tenderer *T*_ideal_^*k*^ is as follows:

(13) Tidealk={max⁡{aijk′},max⁡{bijk′},max⁡{cijk′},…,max⁡{dijk′}}={aijkt,[bijkt−,bijkt+],(cijktL,cijktM,cijktH),…,{[dijktM−,dijktM+],[dijktN−,dijktN+]}},

where *i* = 1,2,…, *M*, *j* = 1,2,…, *N*.

Liu [48] has mentioned an effective method to compare two interval numbers, Li [53] has proposed the center of gravity method for comparing two triangular numbers, and Nayagam and Sivaraman [54] have proposed an effective method to compare two interval-valued intuitionistic fuzzy numbers. In this paper, we will not make a detailed list of these algorithms for concision.

*(c) Distance Calculation*. The following expressions are used to calculate the corresponding distances:

(14) Da=|aijkt−aijk|,Db=|bijkt−−bijk−|+|bijkt+−bijk+|2,Dc=|cijktL−cijkL|+|cijktM−cijkM|+|cijktH−cijkH|3,Dd=((|dijktM−−dijkM−|2+|dijktM+−dijkM+|2+|dijktN−−dijkN−|2+|dijktN+−dijkN+|2)×4−1)1/2,

where *i* = 1,2,…, *M*, *j* = 1,2,…, *N*.

*(d) Gray Relational Coefficient Calculation*. Calculate the gray relational coefficient *ξ*_*ijk*_ of each tenderer from the ideal tenderer *T*_ideal_^*k*^ using the following expression:

(15) $$\begin{array}{c} \xi_{ijk}=\frac{\min\left\{D_{ijk}\right\}+\rho \max\left\{D_{ijk}\right\}}{D_{ijk}+\rho \max\left\{D_{ijk}\right\}}, \end{array}$$

where *D*_*ijk*_ is one of *D*_*a*_, *D*_*b*_, *D*_*c*_, and *D*_*d*_ and *ρ* = 0.5 generally.

*(e) Gray Relational Grade Calculation*. Calculate the gray relational grade *γ*_*ik*_ of each tenderer from the ideal tenderer *T*_ideal_^*k*^ using the following expression:

(16) $$\begin{array}{c} \gamma_{ik}=\overset{N}{\underset{j=1}{\sum }}\omega_{jk}\xi_{ijk}, \end{array}$$

where *ω*_*jk*_ is the weight of evaluation criterion *j* in the perspective of expert *k*, which is obtained in Section 3.1.

*(f) Total Gray Relational Grade Aggregation*. After the previous five steps, we can obtain *K* gray relational grades for each tenderer. After *K* experts evaluate the evaluation criteria of each tenderer, it is necessary to aggregate the *K* gray relational grades of each tenderer. The aggregated gray relational grade *γ*_*i*_ of each tenderer is obtained using the following expression:

(17) $$\begin{array}{c} \gamma_{i}=\overset{K}{\underset{k=1}{\sum }}\omega_{k}\gamma_{ik}, \end{array}$$

where *ω*_*k*_ is the weight of expert *k* in the perspective of government tendering sector, which is obtained in Section 3.1.

*(g) Aggregated Gray Relational Grade Ranking*. By ranking all the *M* aggregated gray relational grades in decreasing order, the optimal tenderer with the largest gray relational grade is identified.

## 4. An Illustrative Example with the Prototype System

In this section, we will illustrate an example for GeT system searching for the optimal tenderer so as to test the practicality and effectiveness of our proposed approach. The software prototype was developed in  .net and ExtJS framework.

**Figure 5:**
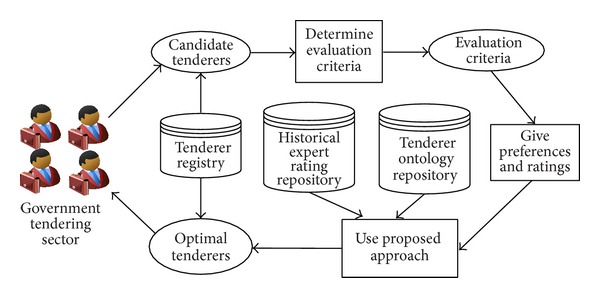
The operational procedure of identifying the optimal tenderer.

The illustrative example displays the identification of an optimal tenderer that has the biggest gray relational grade in a specified context.  Figure 5 shows the operational procedure for identifying the optimal tenderer with our proposed HFAHP-GRA methodology. Firstly, several candidate tenderers are screened out from all the effective tenderers that are saved in the tenderer registry. Secondly, choose or input the evaluation criteria. Thirdly, the government tendering sector gives its preferences on one expert versus the other, and the evaluation experts give their preferences on one evaluation criterion versus the other and their ratings on evaluation criteria of each tenderer. Finally, our proposed approach infers an optimal tenderer that has the biggest gray relational grade from all the candidate tenderers. The historical expert ratings and tenderer information are extracted from historical expert rating repository and tenderer ontology repository, respectively. The tenderer registry is applied to store some related information of tenderers. Our previous works [55, 56] have developed a rich body of OWL-based service ontologies that can provide valid reference for this illustrative example.

**Figure 6:**
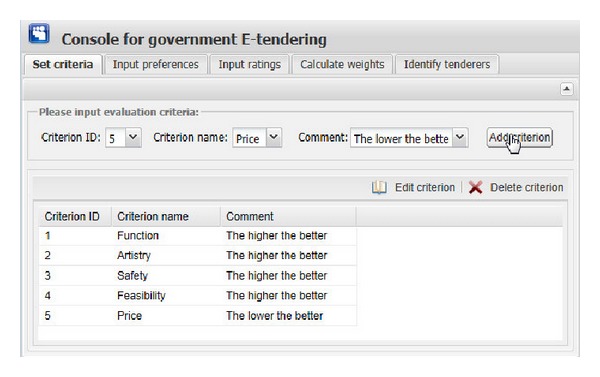
The graphical interface for setting evaluation criteria.

**Figure 7:**
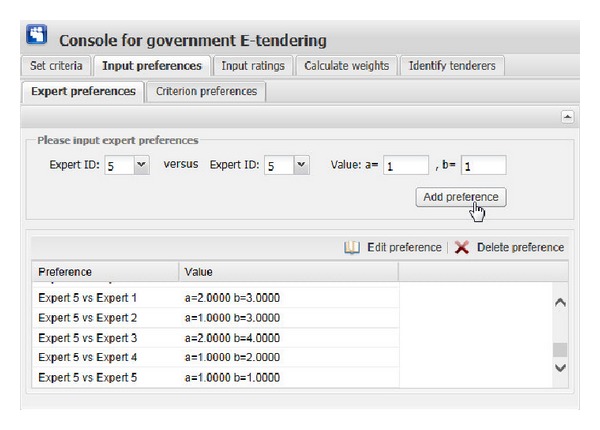
The graphical interface for inputting preferences of government sector and experts.

**Figure 8:**
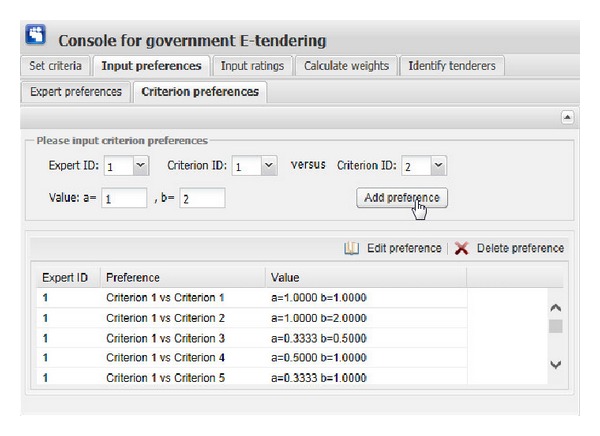
The graphical interface for inputting preferences of experts on criteria.

**Figure 9:**
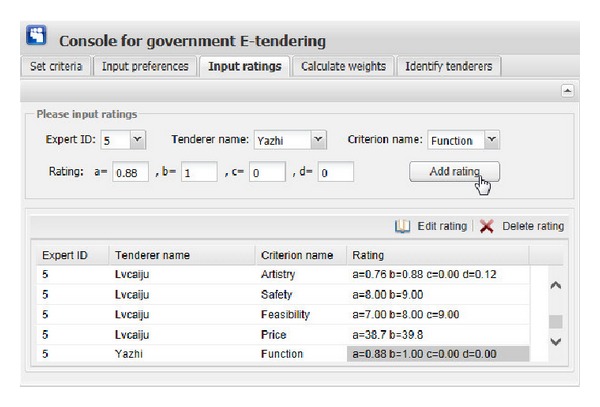
The graphical interface for inputting ratings of experts.

**Figure 10:**
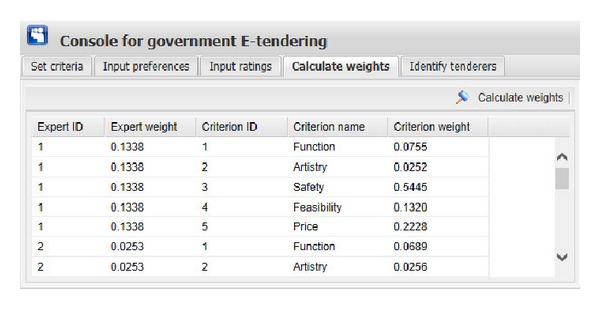
The graphical interface for obtaining weights of experts and evaluation criteria.

**Figure 11:**
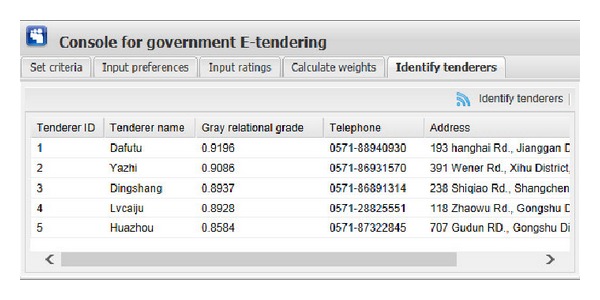
The graphical interface for identifying the optimal tenderers.

Figures 6 to 11 show the graphical interfaces of the process of identifying optimal tenderer in the prototype system. This process is shown as follows.

1. We assume that one governmental department wants to redecorate its whole office block, and the government tendering sector wants to get an appropriate decoration firm through open tendering online. First of all, the government tendering sector needs to set the evaluation criteria (Figure 6), including criterion ID, criterion name, and the comment of criterion (the lower the better or the higher the better). Then, click the “Add criterion” button, and the set evaluation criterion is shown. In this example, there are five evaluation criteria, including “Function,” “Artistry,” “Safety,” “Feasibility,” and “Price.” “Edit criterion” and “Delete criterion” buttons are used to edit or delete the evaluation criteria shown in the window if the criteria are set by mistake.
2. After setting the right evaluation criteria, the preferences of government tendering sector on one expert versus the other should be inputted. The input information consists of expert ID and preference value, which is presented as interval value with Likert numbers as upper bound and lower bound. Then, click the corresponding “Add preference” button in Figure 7, the corresponding preference is shown in the corresponding window. “Edit preference” and “Delete preference” buttons are used to edit or delete preferences shown in the corresponding window if the preferences are wrongly set.
3. Likewise, the preferences of each expert on one evaluation criterion versus the other should be inputted. The input information includes the expert ID, criterion ID, and preference value. Then, click the corresponding “Add preference” button in Figure 8; the corresponding preference is shown in the corresponding window. “Edit preference” and “Delete preference” buttons are used to edit or delete preferences shown in the corresponding window if the preferences are wrongly set.
4. Figure 9 shows the graphical interface for inputting the ratings of each expert on evaluation criteria of each tenderer. The input information consists of expert ID, tenderer name, criterion name, and corresponding rating. During this process, the ratings are expressed as different types of fuzzy numbers, including real number, interval number, triangular number, and interval-valued intuitionistic fuzzy number. In our illustrative example, the evaluation criteria “Function” and “Artistry” are expressed as interval-valued intuitionistic fuzzy numbers, “Safety” is presented as interval numbers, “Feasibility” is expressed as triangular numbers, and “Price” is presented as real numbers or interval numbers. The user of this system only needs to input the corresponding bound values (we use “a,” “b,” “c,” and “d” to present them) of those fuzzy numbers. Interval-valued intuitionistic fuzzy number has four bound values, triangular number has three, interval number has two, and real number has one. After clicking the corresponding “Add rating” button (Figure 9), the corresponding ratings will be shown in the window. “Edit rating” and “Delete rating” buttons are used to edit or delete ratings shown in the window if the ratings are wrongly set.
5. After inputting all the preference information and the rating information and clicking “Obtain weights” button in the top right corner of the window of Figure 10, one can get the weight information of five experts and five evaluation criteria on the perspective of each expert. For example, on the perspective of expert 1, the weights of “Function,” “Artistry,” “Safety,” “Feasibility,” and “Price” are “0.0755,” “0.0252,” “0.5445,” “0.1320,” and “0.2228,” respectively. We can find that, compared with “Artistry,” the other four criteria are much more critical to government in identifying a suitable decoration firm and this just meets the reality.
6. Then, by clicking “Identify tenderers” button in the top right corner of the window of Figure 11, we can get the top five tenderers with their corresponding gray relational grade, telephone number, and address (the related information of tenderers is stored in the tenderer registry). These five optimal tenderers are sorted in the decreasing order according to their corresponding gray relational grades.

## 5. Conclusions

In this paper, we propose a hybridized methodology combining extended fuzzy AHP and modified fuzzy GRA together for government E-tendering to identify the optimal tenderer efficiently and fairly under the circumstance where the ratings of attributes of tenderers are expressed as different kinds of fuzzy numbers and the weight information of experts and evaluation criteria is incompletely known. The main contributions of this paper can be summarized as follows.

- Development of a methodology for web-based bid evaluation of government E-tendering. The hybridized methodology combines fuzzy AHP and fuzzy GRA which are already widely applied in many other fields and confirmed to be effective, but such a combination has not been found in the area of government E-tendering in the literatures.
- Extension of fuzzy AHP-GRA based methodology. We extend the fuzzy AHP-GRA based methodology to hybrid fuzzy area so that different types of vague numbers can be calculated. This extension effectively solves a problem that experts are most likely to express their evaluations on tenderers as numerous kinds of fuzzy numbers. What is more, we assume that the weight information of experts and evaluation criteria is incompletely known. This assumption just suits the reality.
- Development of a prototype system for government E-tendering, which enables better transparency and less costs so as to exploit the superiorities of tendering to the full.

However, our current approach still has limitations. Although there already exist many upper-level ontologies and domain-specific ontologies, few ontologies express the attributes of tenderers as numerous types of fuzzy numbers. Thus, it is urgent to overcome this limitation in our future works so as to reduce the difficulties of putting our proposed approach into practice.

## Figures and Tables

**Table 1:** The standard values of *R*.*I*.

*n*	1	2	3	4	5	6	7	8	9	10

*R*.*I*.	0	0	0.52	0.89	1.12	1.26	1.36	1.41	1.46	1.49

**Table 2:** The relations between linguistic labels and triangular fuzzy number.

Linguistic labels	Triangular fuzzy number
Very poor (VP)	(0.1, 0.2, 0.3)
Poor (P)	(0.2, 0.3, 0.4)
Slightly poor (SP)	(0.3, 0.4, 0.5)
Fair (F)	(0.4, 0.5, 0.6)
Slightly good (SG)	(0.5, 0.6, 0.7)
Good (G)	(0.6, 0.7, 0.8)
Very good (VG)	(0.7, 0.8, 0.9)

## References

[1] Betts M, Black P, Christensen S (2006). Towards secure and legal E-tendering. *Electronic Journal of Information Technology in Construction*.

[2] Chan LS, Chiu DKW, Hung PCK E-tendering with web services: a case study on the tendering process of building construction.

[3] Du TC (2009). Building an automatic e-tendering system on the Semantic Web. *Decision Support Systems*.

[4] Ajam M, Alshawi M, Mezher T (2010). Augmented process model for e-tendering: towards integrating object models with document management systems. *Automation in Construction*.

[5] Du R, Foo E, Nieto JG, Boyd C (2005). Designing secure e-tendering systems. *Trust, Privacy, and Security in Digital Business*.

[6] Mohammadi S, Jahanshahi H A secure e-tendering system.

[7] Eadie R, Perera S, Heaney G (2010). A cross discipline comparison of rankings for e-procurement drivers and barriers within UK construction organisations. *Electronic Journal of Information Technology in Construction*.

[8] Liao TS, Wang MT, Tserng HP (2002). A framework of electronic tendering for government procurement: a lesson learned in Taiwan. *Automation in Construction*.

[9] Singh RK, Benyoucef L (2011). A fuzzy TOPSIS based approach for e-sourcing. *Engineering Applications of Artificial Intelligence*.

[10] Yan Y, Huang L, Guo K, Huang Y (2009). Research and development on web-based decision support system to bid evaluation of agricultural machinery. *Computer Engineering and Design*.

[11] Bar AA, Basili V, Jedaibi WA, Chaudhry AJ (2014). An experience based evaluation process for ERP bids. *International Journal of Web & Semantic Technology*.

[12] Wang DW, Liu XW, Liu LL (2012). Bid evaluation behavior in online procurement auctions involving technical and business experts. *Electronic Commerce Research and Applications*.

[13] Zadeh LA (1965). Fuzzy sets. *Information and Control*.

[14] Zadeh LA (1975). The concept of a linguistic variable and its application to approximate reasoning—I. *Information Sciences*.

[15] Atanassov KT (1986). Intuitionistic fuzzy sets. *Fuzzy Sets and Systems*.

[16] Liu F, Yuan XH (2007). Fuzzy number intuitionistic fuzzy set. *Systems and Mathematics*.

[17] Chen S-M, Tan J-M (1994). Handling multicriteria fuzzy decision-making problems based on vague set theory. *Fuzzy Sets and Systems*.

[18] Hong DH, Choi C-H (2000). Multicriteria fuzzy decision-making problems based on vague set theory. *Fuzzy Sets and Systems*.

[19] Xu ZS, Yager RR (2006). Some geometric aggregation operators based on intuitionistic fuzzy sets. *International Journal of General Systems*.

[20] Wang X-F (2008). Fuzzy number intuitionistic fuzzy geometric aggregation operators and their application to decision making. *Control and Decision*.

[21] Moore RE, Kearfott RB, Cloud MJ (2009). *Introduction To Interval Analysis*.

[22] Xu ZS (2009). A method based on the dynamic weighted geometric aggregation operator for dynamic hybrid multi-attribute group decision making. *International Journal of Uncertainty, Fuzziness and Knowlege-Based Systems*.

[23] Wei GW (2011). Grey relational analysis model for dynamic hybrid multiple attribute decision making. *Knowledge-Based Systems*.

[24] Saaty TL (1980). *The Analytic Hierarchy Process*.

[25] Chan KY, Kwong CK, Dillon TS (2012). An enhanced fuzzy AHP method with extent analysis for determining importance of customer requirements. *Computational Intelligence Techniques for New Product Design*.

[26] Kahraman C, Cebeci U, Ruan D (2004). Multi-attribute comparison of catering service companies using fuzzy AHP: the case of Turkey. *International Journal of Production Economics*.

[27] Erkayman B, Gundogar E, Yilmaz A (2012). An integrated fuzzy approach for strategic alliance partner selection in third-party logistics. *The Scientific World Journal*.

[28] Tam MCY, Tummala VMR (2001). An application of the AHP in vendor selection of a telecommunications system. *Omega*.

[29] Al-Harbi KMA-S (2001). Application of the AHP in project management. *International Journal of Project Management*.

[30] Sun X, Ning P, Tang XL, Yi HH, Zhou LB, Xu XM (2013). Environment risk assessment system for phosphogypsum tailing dams. *The Scientific World Journal*.

[31] Deng JL (1989). Introduction to grey system theory. *The Journal of Grey System*.

[32] Zhang S-F, Liu S-Y (2011). A GRA-based intuitionistic fuzzy multi-criteria group decision making method for personnel selection. *Expert Systems with Applications*.

[33] Wu DS (2009). Supplier selection in a fuzzy group setting: a method using grey related analysis and Dempster-Shafer theory. *Expert Systems with Applications*.

[34] Chen W-H, Tsai M-S, Kuo H-L (2005). Distribution system restoration using the hybrid fuzzy-grey method. *IEEE Transactions on Power Systems*.

[35] Jiang BC, Tasi S-L, Wang C-C (2002). Machine vision-based gray relational theory applied to IC marking inspection. *IEEE Transactions on Semiconductor Manufacturing*.

[36] Lin C-T, Chang C-W, Chen C-B (2006). The worst ill-conditioned silicon wafer slicing machine detected by using grey relational analysis. *International Journal of Advanced Manufacturing Technology*.

[37] Kuo YY, Yang TH, Huang G-W (2008). The use of grey relational analysis in solving multiple attribute decision-making problems. *Computers & Industrial Engineering*.

[38] Beshkooh M, Afshari MA (2012). Selection of the optimal portfolio investment in stock market with a hybrid approach of hierarchical analysis (AHP) and grey theory analysis (GRA). *Journal of Basic and Applied Scientific Research*.

[39] Yang C-C, Chen B-S (2006). Supplier selection using combined analytical hierarchy process and grey relational analysis. *Journal of Manufacturing Technology Management*.

[40] Peng J (2012). Research on the optimization of green suppliers based on AHP and GRA. *Journal of Information and Computational Science*.

[41] Pophali GR, Chelani AB, Dhodapkar RS (2011). Optimal selection of full scale tannery effluent treatment alternative using integrated AHP and GRA approach. *Expert Systems with Applications*.

[42] Salardini F (2013). An AHP-GRA method for asset allocation: a case study of investment firms on Tehran Stock Exchange. *Decision Science Letters*.

[43] Lahby M, Adib A Network selection mechanism by using M-AHP/GRA for heterogeneous networks.

[44] Liu YL, Zhou X, Ren SS, Yang L, Ci S Peer selection in mobile P2P networks based on AHP and GRA.

[45] Liu T The application of fuzzy analytic hierarchy process and grey relational analysis in the taxi passenger satisfaction evaluation.

[46] Samvedi A, Jain V, Chan FTS (2012). An integrated approach for machine tool selection using fuzzy analytical hierarchy process and grey relational analysis. *International Journal of Production Research*.

[47] Gumus AT, Yesim Yayla A, Çelik E, Yildiz A (2013). A combined fuzzy-AHP and fuzzy-GRA methodology for hydrogen energy storage method selection in Turkey. *Energies*.

[48] Liu F (2009). Acceptable consistency analysis of interval reciprocal comparison matrices. *Fuzzy Sets and Systems*.

[49] Xu ZS, Wei CP (1999). A consistency improving method in the analytic hierarchy process. *European Journal of Operational Research*.

[50] Liu F, Zhang W-G, Fu J-H (2012). A new method of obtaining the priority weights from an interval fuzzy preference relation. *Information Sciences*.

[51] Wang Z-J, Li KW (2012). Goal programming approaches to deriving interval weights based on interval fuzzy preference relations. *Information Sciences*.

[52] Xu ZS, Chen J (2008). Some models for deriving the priority weights from interval fuzzy preference relations. *European Journal of Operational Research*.

[53] Li DF (2003). *Fuzzy Multiobjective Many-Person Decision Makings and Games*.

[54] Nayagam VLG, Sivaraman G (2011). Ranking of interval-valued intuitionistic fuzzy sets. *Applied Soft Computing Journal*.

[55] Zhang WY, Yin JW (2009). Weaving a semantic grid for multidisciplinary collaborative design. *International Journal of Production Research*.

[56] Cai M, Zhang WY, Zhang K (2011). ManuHub: a semantic web system for ontology-based service management in distributed manufacturing environments. *IEEE Transactions on Systems, Man, and Cybernetics A: Systems and Humans*.

